# Supplementary material for: Localized heme sensing through a ternary molecular glue
Source: bioRxiv. 2026 May 8:2026.05.07.723605. Preprint. [Version 1] doi: 10.64898/2026.05.07.723605 (PMC13174492; doi:10.64898/2026.05.07.723605)
Supplement: Supplement 1 [file NIHPP2026.05.07.723605v1-supplement-1.pdf]

bioRxiv preprint doi: https://doi.org/10.64898/2026.05.07.723605; this version posted May 8, 2026. The copyright holder for this preprint (which was not certified by peer review) is the author/funder, who has granted bioRxiv a license to display the preprint in perpetuity. It is made available under aCC-BY 4.0 International license.

Supplementary Figure legends

Figure S1: CUL2^{FEM1B} is functionally linked to heme metabolism. Related to Figure 1.

A. FEM1B promotes AML cell proliferation. mCherry-expressing *ΔFEM1B* MOLM13 cells were mixed with GFP-expressing WT cells and followed by flow cytometry for 12d. Datapoints represent mean ± S.E.M. of n=3 independent experiments. **B.** Mitochondrial processes and components are essential in the MV4-11 cell line used in the synthetic lethality CRISPR screen. Essential genes in MV4-11 WT cells were determined using combined CasTLE analysis of differential sgRNA abundance at d0, d12 and d21 of the whole genome CRISPR screen presented in Figure 1B. Gene list enrichment analysis of <5% FDR scoring depleted genes (n=504 genes) was performed using Enrichr [88]. **C.** Whole genome dropout CRISPR screen in THP1 cells with low mitochondrial activity does not reveal ties to heme biosynthesis. Combined CasTLE analysis of change in sgRNA abundance over 30 days in two distinct *ΔFEM1B* THP1 clones compared to WT THP1 cells; 10% FDR. **D.** Mitochondrial genes are not enriched among top essential pathways in THP1 cells. Essential genes in THP1 wt cells were determined using a combined CasTLE analysis of differential sgRNA abundance at d0, d21 and d30 of the whole genome CRISPR screen presented in Figure S1C. Gene list enrichment analysis of n=504 highest scoring depleted genes was performed using Enrichr.

Figure S2: CUL2^{FEM1B} drives heme-dependent degradation of BACH1. Related to Figure 2.

A. FEM1B binds BACH1. Immunoprecipitation of ^{3XFLAG}FEM1B or ^{3XFLAG}FEM1B$^{R126A/L597A}$ from THP1 cells coupled with mass spectrometry identifies BACH1 as a candidate substrate of CUL2^{FEM1B}. **B.** BACH1 degradation depends on FEM1B. Stability reporters for selected FEM1B binders were transiently expressed in WT or *ΔFEM1B* HEK293T cells in the absence or presence

bioRxiv preprint doi: https://doi.org/10.64898/2026.05.07.723605; this version posted May 8, 2026. The copyright holder for this preprint (which was not certified by peer review) is the author/funder, who has granted bioRxiv a license to display the preprint in perpetuity. It is made available under aCC-BY 4.0 International license.

of overexpressed FEM1B. The GFP/mCherry ratio was determined using flow cytometry. Data represented as median ± S.E.M. of median fluorescence intensity ratios (MFI) of n=3-4 independent experiments. **C.** Dominant negative CUL2 stabilizes the BACH1 stability reporter in HEK293T cells. Similar results in n=2 independent experiments. **D.** Inhibition of Cullin-Ring ligases or proteasomes, but not lysosomes, stabilizes BACH1. BACH1 stability reporters were expressed in wt HEK293T cells, carfilzomib (2 μM), bafilomycin A (700 nM) were added for 6h, and MLN4924 (1μM) was added for 16h. Similar results in n=2 independent experiments. **E.** $CUL2^{FEM1B}$ does not target BACH2. BACH2 stability reporters were expressed in WT or *ΔFEM1B* HEK293T cells in the absence or presence of FEM1B. Similar results in n=2 independent experiments. **F.** $CUL2^{FEM1A}$ does not target BACH1. BACH1 stability reporters were expressed in *ΔFEM1B* HEK293T cells in the absence or presence of FEM1B or FEM1A. Similar results in n=2 independent experiments. **G.** Degradation of a BACH1 stability reporter induced by hemin depends entirely on FEM1B in AML cells. BACH1 stability reporters composed of brighter BACH1-mNeonGreen-P2A-mScarlet3 were lentivirally expressed in WT and *ΔFEM1B* MV4-11 cells. Cells were treated with 10μM hemin for 16h. Similar results in n=2 independent experiments. **H.** Iron chelation stabilizes BACH1. BACH1 stability reporters were expressed in WT or *ΔFEM1B* HEK293T cells, which were treated with 1mM DFP for 16h as indicated. Similar results in n=2 independent experiments. **I.** Hemin does not induce degradation of other FEM1B substrates. FNIP1 and COA4 stability reporters were expressed in WT or *ΔFEM1B* HEK293T cells, and cells were treated with 10μM hemin for 16h. Similar results in n=2 independent experiments. **J.** FBXO22 and FBXL17 do not drive heme-induced degradation of BACH1. BACH1 stability reporters were expressed in *ΔFEM1B* HEK293T cells together with FEM1B, FBXO22 or FBXL17, and cells were treated with 10μM hemin for 16h, as indicated. Similar results in n=2 independent experiments. **K.** Depletion of FBXO22 or FBXL17 does not inhibit heme-induced degradation of BACH1 in AML cells. FBXO22 or FBXL17 were depleted from THP1 (left) or MOLM13 (right) cells stably expressing a BACH1 stability reporter. Cells were treated with 10μM hemin for 16h. Similar

bioRxiv preprint doi: https://doi.org/10.64898/2026.05.07.723605; this version posted May 8, 2026. The copyright holder for this preprint (which was not certified by peer review) is the author/funder, who has granted bioRxiv a license to display the preprint in perpetuity. It is made available under aCC-BY 4.0 International license.

results in n=2 independent experiments. **L.** Heme-induced degradation of endogenous BACH1 is not affected by depletion of FBXO22. HEK293T cells were depleted of FBXO22 by two distinct sgRNAs. Cells were treated with 10µM hemin for 16h, and endogenous BACH1 levels were assessed by Western blotting. Similar results in n=2 independent experiments. **M.** Depletion of the heme biosynthesis enzyme ALAD stabilizes BACH1 dependent on FEM1B. ALAD was depleted from WT or *ΔFEM1B* HEK293T cells stably expressing a BACH1 stability reporter. Similar results in n=3 independent experiments. **N.** Increasing endogenous heme destabilizes BACH1 dependent on FEM1B. BACH1 stability reporters were expressed in HEK293T cells together with vectors for expression of ALAS1 (left) or ALAD (right). Similar results in n=2 independent experiments. **O.** Disruption of heme integration into ETC cIV destabilizes BACH1 dependent on FEM1B and heme levels. COX10 (left) or COX15 (right) were co-depleted with ALAS1 from WT HEK293T cells stably expressing the BACH1 stability reporter. Similar results in n=2 independent experiments. **P.** Disruption of ETC cIV assembly destabilizes BACH1. cIV assembly factors were depleted from WT HEK293T cells stably expressing the BACH1 stability reporter. Similar results in n=2 independent experiments.

Figure S3: $CUL2^{FEM1B}$ recognizes a specific Cys/Pro motif in BACH1. Related to Figure 3.

A. Domain map of BACH1 with Cys/Pro motifs (CP) highlighted. **B.** Mutation of the C-terminal CP motif disrupts heme-induced BACH1 degradation through $CUL2^{FEM1B}$. BACH1 stability reporters were expressed in WT and *ΔFEM1B* HEK293T cells. Cells were treated with 10µM hemin for 16h. Similar results in n=2 independent experiments. **C.** Deletion or mutation of the N-terminal BTB-domain does not affect heme-induced BACH1 degradation. BACH1 stability reporters were expressed in WT HEK293T cells together with FEM1B or FBXO22. Cells were treated with 10µM hemin for 16h as indicated. Similar results in n=2 independent experiments.

bioRxiv preprint doi: https://doi.org/10.64898/2026.05.07.723605; this version posted May 8, 2026. The copyright holder for this preprint (which was not certified by peer review) is the author/funder, who has granted bioRxiv a license to display the preprint in perpetuity. It is made available under aCC-BY 4.0 International license.

Figure S4: Cryo-EM processing workflow. Related to Figure 3.

A. Cryo-EM data processing workflow of the CUL2-RBX1-ELOB-ELOC-FEM1B-BACH1^{CT} complex in dimeric conformation (EMD-76876). **B.** Cryo-EM data processing workflow of the CUL2-RBX1-ELOB-ELOC-FEM1B-BACH1^{CT} complex in monomeric conformation (EMD-76897) and local refinement of the FEM1B-BACH1 interface region (EMD-76910) highlighted in dashed line circle in the monomeric conformation map.

Figure S5: CUL2^{FEM1B} binds BACH1 at its mitochondrial anchor site. Related to Figure 3.

A. Size exclusion chromatogram of the CUL2-RBX1-ELOB-ELOC-FEM1B-BACH1^{CT} complex. The complex elutes at the volume expected for a dimeric E3 ligase. Collected fractions indicated by a dashed line. Similar results in n>5 independent experiments. **B.** Representative SDS-PAGE analysis of collected fractions reveals a fully assembled complex. (*) denotes fractions collected for structural analysis. Similar results in n>5 independent experiments. **C.** Mutation of FEM1B residues involved in homodimerization (4D) disrupt its ability to induce BACH1 degradation. FEM1B WT or 4D-mutant (F549D/V584D/I587D/L588D) and BACH1 stability reporters were expressed in *ΔFEM1B* HEK293T cells, and cells were treated with 10μM hemin for 16h. Similar results in n=2 independent experiments. **D.** BACH1-bound CUL2^{FEM1B} complex frozen on carbon coated EM-grids yields a monomeric structure. Cryo-EM density map of the CUL2-RBX1-ELOB/C-FEM1B-BACH1^{CT} complex, EMD-76897, contour level 0.052) **E.** Surface representation of the complex shown in (D.) reveals structural details of complex assembly and highlights positioning of BACH1 at the flexible N-terminus of FEM1B (PDB 12ZS). Same colors used as in Figure 3E. Right: 45° rotation to the right. **F.** BACH1 and TOM20 both bind the cap of the FEM1B N-terminal ankyrin repeats. Left: Cryo-EM guided AF3 model of the FEM1B-BACH1 interface. Right: FEM1B-TOM20 interface from the published locally refined structure (PDB 9JCE) [61]. **G.**

bioRxiv preprint doi: https://doi.org/10.64898/2026.05.07.723605; this version posted May 8, 2026. The copyright holder for this preprint (which was not certified by peer review) is the author/funder, who has granted bioRxiv a license to display the preprint in perpetuity. It is made available under aCC-BY 4.0 International license.

Dimerization of BACH1 allows additional interaction with the backside of the FEM1B N-terminus. FEM1B-BACH1 interface highlighting residues L630/Q634 involved in BACH1 dimerization and residues mediating interactions at the backside of FEM1B (BACH1 Y641 and FEM1B K60). **H.** Modeling the extension of BACH1 helices places the lysine rich DNA-binding domains close to RBX1 *in trans*. BACH1 lysine residues in proximity to RBX1 are shown in red. **I.** Depletion of endogenous heme biosynthetic enzymes does not affect BACH1 stability if heme binding has been obliterated. ALAD, or HMOX2 were depleted from WT HEK293T cells stably expressing a $BACH1^{C646S}$ stability reporter. Similar results in n=2 independent experiments. **J.** Increasing endogenous heme does not affect mutant BACH1 degradation. $BACH1^{C646S}$ stability reporters were expressed in HEK293T cells together with ALAS1 or ALAD, as indicated. Similar results in n=2 independent experiments. **K.** Mutation of residues involved in dimerization of $BACH1^{CT}$ renders BACH1 insensitive to FEM1B-mediated and heme induced degradation. $BACH1^{L630A}$ or $BACH1^{Q634E}$ stability reporters were expressed in WT or *ΔFEM1B* HEK293T cells, and cells were treated with 10μM hemin for 16h. Similar results in n=2 independent experiments. Stability of $BACH1^{wt}$ under these conditions is shown in Figure 3K. **L.** Mutation of FEM1B residues at the N-terminal helix disrupt BACH1 degradation. FEM1B variants and BACH1 stability reporters were expressed in *ΔFEM1B* HEK293T cells and cells were treated with 10μM hemin for 16h. Similar results in n=3 independent experiments. **M-N.** Mutation of residues at the backside FEM1B surface recruiting the second $BACH1^{CT}$ dimer subunit attenuate heme-induced degradation of BACH1. WT-FEM1B or $FEM1B^{K60A}$ variants and/or WT-BACH1 or $BACH1^{Y641A}$ stability reporters were expressed in HEK293T cells, which were treated with 10μM hemin for 16h. Similar results in n=2 independent experiments. **O.** FEM1B residues in the central grove required for binding other substrates or regulators are not required for BACH1 degradation. FEM1B variants and BACH1 stability reporters were expressed in *ΔFEM1B* HEK293T cells, which were treated with 10μM hemin for 16h. Similar results in n=2 independent experiments.

bioRxiv preprint doi: https://doi.org/10.64898/2026.05.07.723605; this version posted May 8, 2026. The copyright holder for this preprint (which was not certified by peer review) is the author/funder, who has granted bioRxiv a license to display the preprint in perpetuity. It is made available under aCC-BY 4.0 International license.

Figure S6: Heme acts as a ternary molecular glue. Related to Figure 4.

A. FEM1B residues involved in BACH1 binding are highly conserved but specific to FEM1B. Multiple sequence alignment of FEM1A, FEM1B and FEM1C and conservation scores of FEM1B shown for N-terminal FEM1B regions involved in BACH1 binding. Critical residues validated in this study highlighted in red boxes. **B-C.** Binding of FEM1B to BACH1^{CT} requires heme as a molecular glue. Biolayer interferometry using streptavidin tips bound to Biotin-FEM1B were incubated with increasing concentrations of BACH1^{CT} in the presence (B.) or absence (C.) of 20μM Hemin and association/dissociation were monitored over time. Sensorgrams were fit to a 2:1 heterogeneous ligand binding model and fitted curves are overlaid with experimental data. The apparent K_D in the presence of 20 μM hemin was determined using steady-state analysis. Similar results in n=2 independent experiments. **D-E.** Apparent dissociation constant for the interaction of Biotin-BACH1^{CT} and FEM1B (D.) or Biotin-FEM1B and BACH1^{CT} (E.) in the presence of 20 μM hemin was determined by plotting the equilibrium response as a function of ligand concentration and fitting to a steady-state binding isotherm. The apparent K_D values are reported as best fit ± standard error of the steady-state fit. **F.** Binding of BACH1^{CT} to FEM1B requires heme as a molecular glue. Recombinant BACH1^{CT} was subjected to pulldown assays with MBP-FEM1B immobilized to amylose resin with increasing concentrations of hemin. Interaction was detected by Coomassie staining of SDS-PAGE gels or Western blotting. Similar results in n=2 independent experiments. **G.** Ubiquitylation of BACH1^{CT} by CUL2^{FEM1B} shows a dose-dependent requirement for heme. *In vitro* ubiquitylation assays of recombinant BACH1^{CT} by NEDD8-modified CUL2^{FEM1B} with E1, UBE2R1 and ubiquitin probed for BACH1 ubiquitylation by Western blotting against BACH1^{CT}. Similar results in n=2 independent experiments. **H-I.** Interaction of endogenous BACH1 with FEM1B in AML cells depends on heme. BACH1^{3XFLAG} endogenously edited THP1 (H.) or MV4-11 (I.) cells were lysed in the presence of increasing

bioRxiv preprint doi: https://doi.org/10.64898/2026.05.07.723605; this version posted May 8, 2026. The copyright holder for this preprint (which was not certified by peer review) is the author/funder, who has granted bioRxiv a license to display the preprint in perpetuity. It is made available under aCC-BY 4.0 International license.

concentrations of hemin and subjected to immunoprecipitation and western blotting with the indicated antibodies. Similar results in n=2 independent experiments. **J.** Purified FEM1B-BACH1 complex contains heme. ^{3XFLAG}FEM1B and BACH1^{HA} were transiently expressed in Expi293F cells and lysates subjected to αFLAG-immunoprecipitation, The concentration of heme in eluted protein complexes was determined using an apo-peroxidase based assay. Technical replicates represented as as mean ± SD. Similar results in n=2 independent experiments. Statistical significance was determined using a two-tailed Student's t-test (* p<0.1). **K.** Purified FEM1B-BACH1 complex contains iron. Eluted proteins obtained as described above were further digested with chymotrypsin and denatured with HNO_3. Samples were then probed for several transition metals using inductively coupled plasma spectroscopy. Technical replicates represented as mean ± SD. Similar results in n=2 independent experiments. **L.** Mutation of critical FEM1B residues abrogates heme-induced BACH1 binding *in vitro*. Recombinant BACH1^{CT} was subjected to pulldown assays with MBP-FEM1B mutants immobilized to amylose resin ± 20μM hemin. Interaction was detected by Coomassie staining of SDS-PAGE gels or western blotting with a BACH1-antibody. Similar results in n=2 independent experiments. **M.** Loss of FEM1B interacting carboxy groups in heme disrupts BACH1-FEM1B binding *in vitro*. Recombinant BACH1^{CT} was subjected to pulldown assays with MBP-FEM1B mutants immobilized to amylose resin in the presence of 20μM heme or heme analogues. Interaction was detected by Coomassie staining of SDS-PAGE gels or western blotting with a BACH1-antibody. Similar results in n=2 independent experiments. **N.** Mutation of critical BACH1 residues abrogates heme-induced FEM1B binding. BACH1^{wt}, BACH1$^{L617A/L620A}$ or BACH1^{C646S} (all BACH1^{CT}) were subjected to pulldown assays with MBP-FEM1B immobilized to amylose resin ± 20μM hemin. Interaction was detected by Coomassie staining of SDS-PAGE gels or Western blotting with a BACH1-antibody. Similar results in n=2 independent experiments.

bioRxiv preprint doi: https://doi.org/10.64898/2026.05.07.723605; this version posted May 8, 2026. The copyright holder for this preprint (which was not certified by peer review) is the author/funder, who has granted bioRxiv a license to display the preprint in perpetuity. It is made available under aCC-BY 4.0 International license.

Figure S7: Mutation of critical BACH1 or FEM1B residues in cells disrupts binding and heme-induced degradation. Related to Figure 4.

A-B. Mutation of BACH1/FEM1B residues abrogates heme-mediated interaction in cells. ^{3XFLAG}FEM1B (A.) or BACH1^{3XFLAG} (B.) variants were expressed in HEK293T cells and immunoprecipitates were analyzed by Western blotting with the indicated antibodies. Lysis and IP were performed ± 20μM Hemin as indicated. Similar results in n=2 independent experiments. **C.** Mutation of the heme binding residue C646 in BACH1 disrupts binding to CUL2^{FEM1B}, while the interaction with known key interactors, such as MAF proteins, remains intact. BACH^{wt} or BACH1^{C646S} (both 3X-FLAG) were lentivirally expressed in MV4-11 cells and immunoprecipitated proteins were detected by mass spectrometry. Data of n=3 technical MS-replicates shown. **D.** Sanger sequencing confirms correct CRISPR editing at the endogenous BACH1 locus. **E.** Mutation of critical residues at the endogenous BACH1 locus renders it insensitive to heme induced degradation. MV4-11 cells were endogenously edited using CRISPR-Cas9 and homology directed repair, treated with 10μM hemin for 16h, and lysates subjected to Western blot analysis. Similar results in n=2 independent experiments.

Figure S8: Ternary glue signaling elicits a gene expression response to alleviate heme toxicity. Related to Figure 5.

A. BACH1 and FEM1B co-regulate HMOX1 expression. BACH1 or FEM1B were depleted in MOLM13 cells using specific shRNAs and HMOX1 expression was analyzed by qPCR. Data of n=4 independent experiments represented as mean ± SD. **B.** FEM1B and BACH1 knockdown verification of MV4-11 and MOLM13 qPCR experiments shown in Figures 5C, 5E and S8A, S8D. Data of n=4-5 independent experiments represented as mean ± SD. **C.** Enhanced BACH1 binding to HMOX1 enhancer upon FEM1B loss. *ΔFEM1B* and wt MV4-11 cells were subjected to BACH1

bioRxiv preprint doi: https://doi.org/10.64898/2026.05.07.723605; this version posted May 8, 2026. The copyright holder for this preprint (which was not certified by peer review) is the author/funder, who has granted bioRxiv a license to display the preprint in perpetuity. It is made available under aCC-BY 4.0 International license.

CHIP and qPCR of known BACH1 binding sites in the EN1 enhancer of HMOX [89]. Heme treated cells that degrade BACH1 served as control. Data expressed as percentage of input. Similar results observed in n=3 independent experiments. **D.** Metallothionein gene expression is impacted by BACH1- and/or FEM1B-depletion. BACH1 and/or FEM1B were depleted in MV4-11 cells using specific shRNAs and expression of MT1F, MT1G, MT1X or MT2A were monitored by qPCR. Data of n=4-5 independent experiments represented as mean ± SD. **E.** BACH1 and TOM20 compete for binding to FEM1B. Recombinant $BACH1^{CT}$ was subjected to pulldown assays with MBP-FEM1B immobilized to amylose resin in the presence of 20μM hemin and increasing concentrations of the FEM1B-binding cytosolic domain of TOM20 lacking flexible Lys residues ($TOM20^{62\text{-}127}$) [36]. Interaction was detected by Coomassie staining. Similar results in n=2 independent experiments. **F.** Ubiquitylation of $BACH1^{CT}$ by $CUL2^{FEM1B}$ is impeded by TOM20. *In vitro* ubiquitylation assays of $BACH1^{CT}$ by NEDD8-modified $CUL2^{FEM1B}$ complexes with E1, UBE2R1 and ubiquitin and 20μM hemin. Increasing concentrations of $TOM20^{62\text{-}127}$ were added as indicated. BACH1 ubiquitylation was detected by Western blotting. Similar results in n=2 independent experiments. **G.** TOM20 overexpression stabilizes BACH1 in cells. BACH1 stability reporters were expressed in HEK293T cells together with TOM20 or cytoplasmic $TOM20^{25\text{-}145}$ as indicated. Similar results in n=2 independent experiments. **H.** Anchoring all FEM1B to mitochondria inhibits BACH1 degradation. BACH1 or FNIP1-mito reporter constructs were transiently transfected into *ΔFEM1B* HEK293T cells together with control, $FEM1B^{wt}$ or FEM1B constructs engineered to be anchored to the outer mitochondrial membrane. The N-terminal anchor helix (residues 1-30) of the OMM protein AKAP1 was appended to the FEM1B N-terminus separated by GGGS-linkers of different lengths to induce mitochondrial tethering. Similar results in n=2 independent experiments. Statistical significance in (A-D.) was determined using one-sample t-tests and two-tailed Student's t-tests (* p<0.1, ** p<0.01, *** p<0.001 and **** p<0.0001).

bioRxiv preprint doi: https://doi.org/10.64898/2026.05.07.723605; this version posted May 8, 2026. The copyright holder for this preprint (which was not certified by peer review) is the author/funder, who has granted bioRxiv a license to display the preprint in perpetuity. It is made available under aCC-BY 4.0 International license.

Figure S9: Loss of FEM1B sensitizes AML cells to the BCL2-inhibitor Venetoclax. Related to Figure 6.

A. Low expression of the representative heme biosynthesis enzymes CPOX and FECH is conserved over all AML subtypes. Gene expression analysis of patients and controls described above separated by genetically defined subtypes according to the WHO 2022 classification. Individual datapoints shown together with mean. **B.** Cell competition assays confirm strong synergism between Venetoclax and loss of FEM1B. mCherry-expressing *ΔFEM1B* and GFP-expressing WT MV4-11 cells were mixed and incubated with the indicated concentrations of Venetoclax for 4 days, after which mCherry/GFP ratios were determined by flow cytometry. Datapoints represent n=3 independent experiments. **C-D.** Acute FEM1B depletion also sensitizes AML cells to Venetoclax treatment. mCherry- or GFP-expressing MV4-11 (C.) or MOLM13 (D.) were infected with sgCTRL or two distinct sgRNAs targeting FEM1B, respectively. Following selection with puromycin, cells were mixed and treated with the indicated concentrations of Venetoclax for 4 days, after which mCherry/GFP ratios were determined by flow cytometry. Datapoints represent n=2-4 independent experiments. **E-H.** Loss of FEM1B sensitizes AML cells to a range of BCL family inhibitors. mCherry-expressing *ΔFEM1B* and GFP-expressing WT MV4-11 cells were mixed and incubated with the indicated concentrations of ABT-737 (E.), Navitoclax/ABT-263 (F.), S55746 (G.) or S63845 (H.) for 4 days, after which mCherry/GFP ratios were determined by flow cytometry. Datapoints represent n=2-3 independent experiments. **I.** *ΔFEM1B* AML cells show increased apoptosis in upon Venetoclax treatment. *ΔFEM1B* and WT MV4-11 cells were treated with the indicated concentrations of Venetoclax for 8 hours and the fraction of early apoptotic (Annexin+/PI-) and late apoptotic (Annexin+/PI+) cells was determined by flow cytometry. Apoptosis was inhibited by addition of 50µM Z-VAD-FMK as indicated. Data expressed as mean ± SD of n=3 independent experiments. **J.** Caspase inhibition rescues

bioRxiv preprint doi: https://doi.org/10.64898/2026.05.07.723605; this version posted May 8, 2026. The copyright holder for this preprint (which was not certified by peer review) is the author/funder, who has granted bioRxiv a license to display the preprint in perpetuity. It is made available under aCC-BY 4.0 International license.

synthetic lethality of Venetoclax in *ΔFEM1B* AML cells. mCherry-expressing *ΔFEM1B* and GFP-expressing WT MV4-11 cells were mixed and incubated with the indicated concentrations of Venetoclax and 50μM of Z-VAD-FMK for 2 days, after which mCherry/GFP ratios were determined by flow cytometry. Datapoints represent n=2-3 independent experiments.

bioRxiv preprint doi: https://doi.org/10.64898/2026.05.07.723605; this version posted May 8, 2026. The copyright holder for this preprint (which was not certified by peer review) is the author/funder, who has granted bioRxiv a license to display the preprint in perpetuity. It is made available under aCC-BY 4.0 International license.

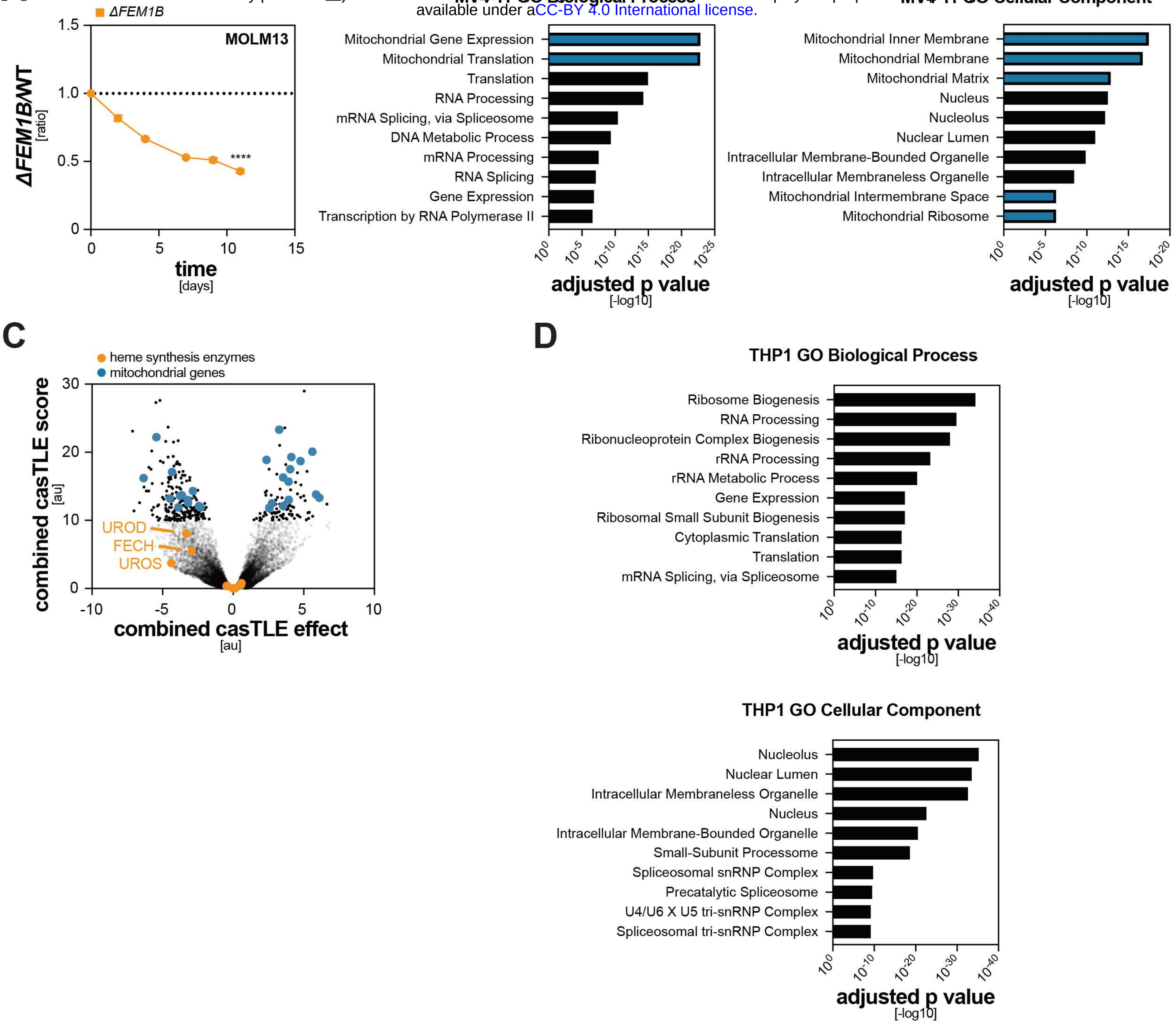

Figure S1

bioRxiv preprint doi: https://doi.org/10.64898/2026.05.07.723605; this version posted May 8, 2026. The copyright holder for this preprint (which was not certified by peer review) is the author/funder, who has granted bioRxiv a license to display the preprint in perpetuity. It is made available under aCC-BY 4.0 International license.

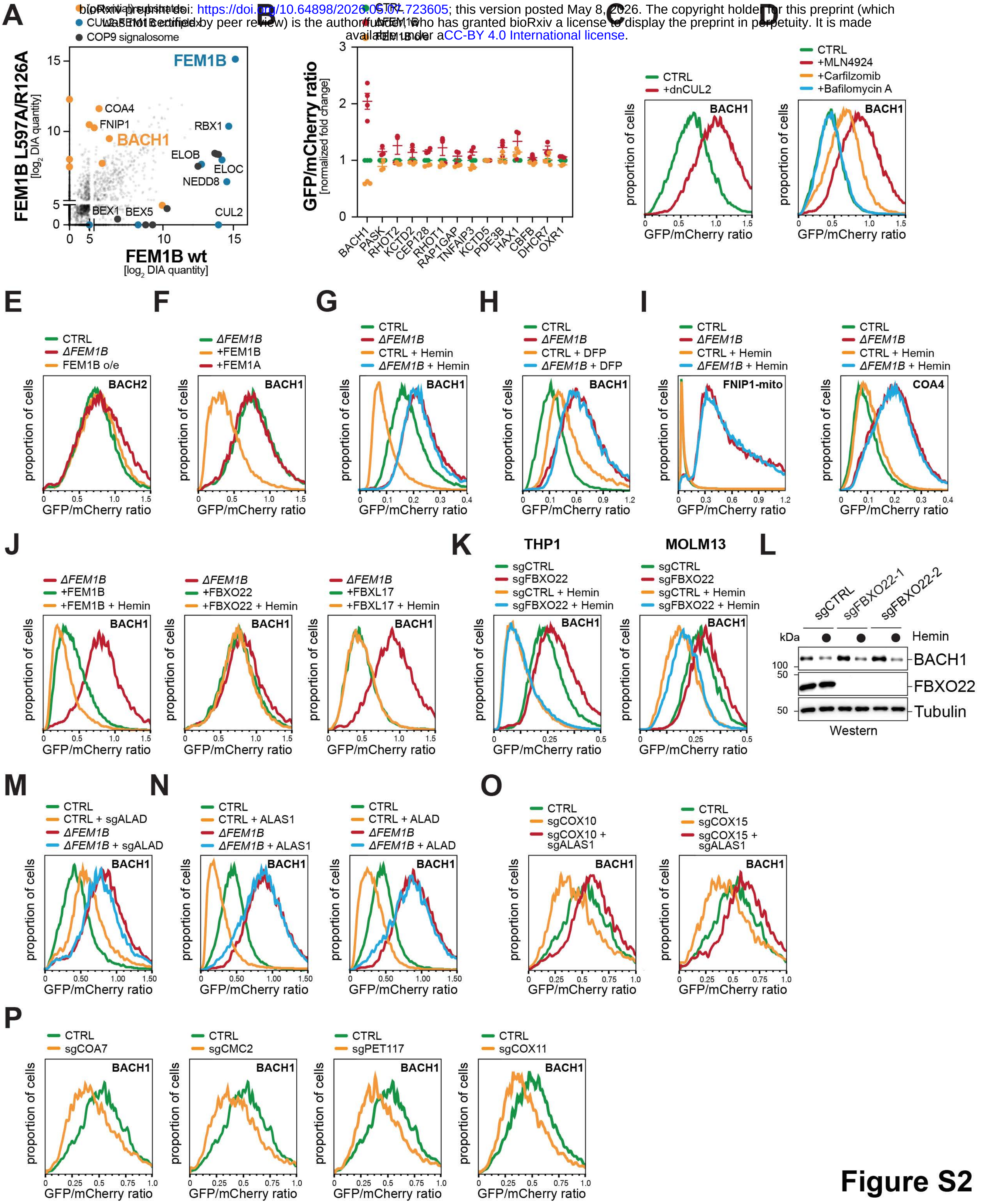

Figure S2

bioRxiv preprint doi: https://doi.org/10.64898/2026.05.07.723605; this version posted May 8, 2026. The copyright holder for this preprint (which was not certified by peer review) is the author/funder, who has granted bioRxiv a license to display the preprint in perpetuity. It is made available under aCC-BY 4.0 International license.

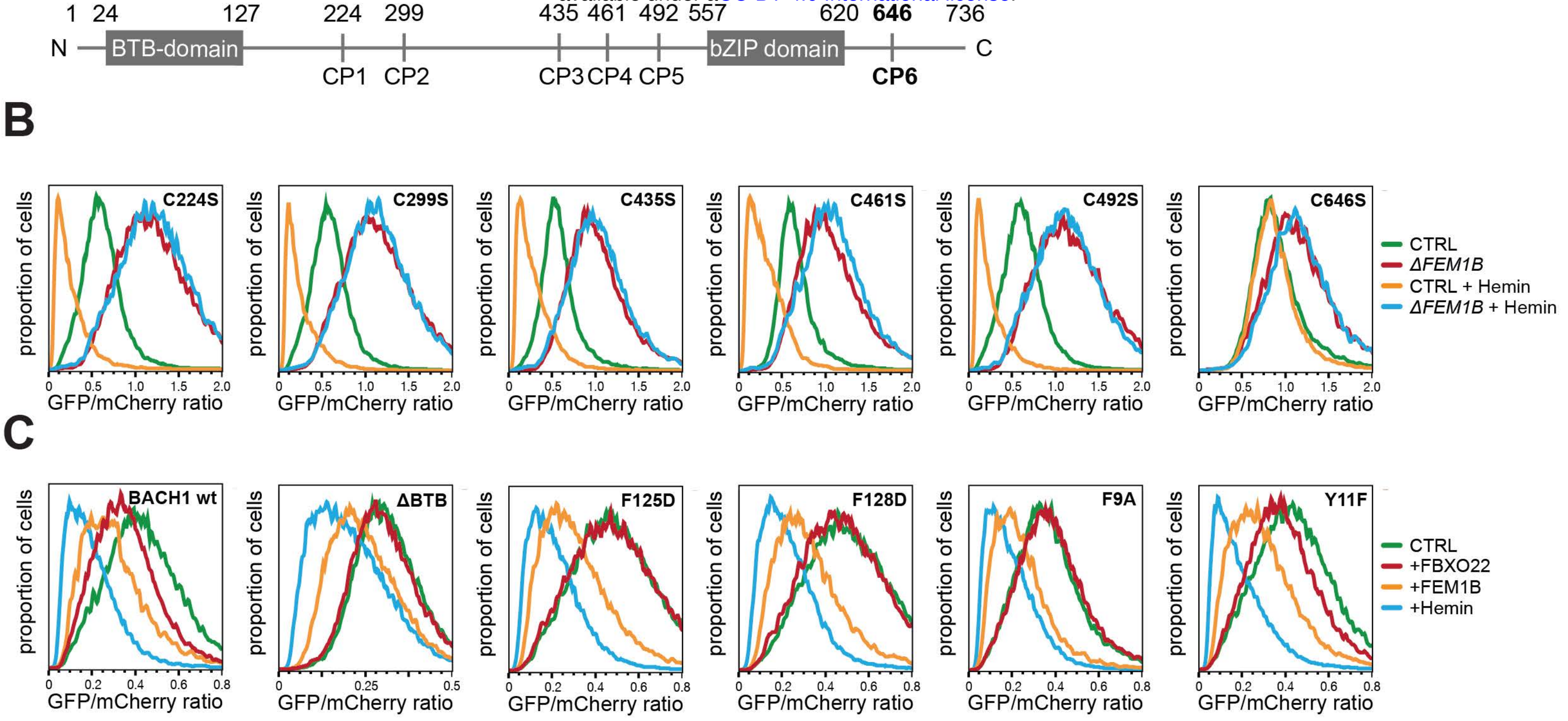

Figure S3

bioRxiv preprint doi: https://doi.org/10.64898/2026.05.07.723605; this version posted May 8, 2026. The copyright holder for this preprint (which was not certified by peer review) is the author/funder, who has granted bioRxiv a license to display the preprint in perpetuity. It is made available under aCC-BY 4.0 International license.

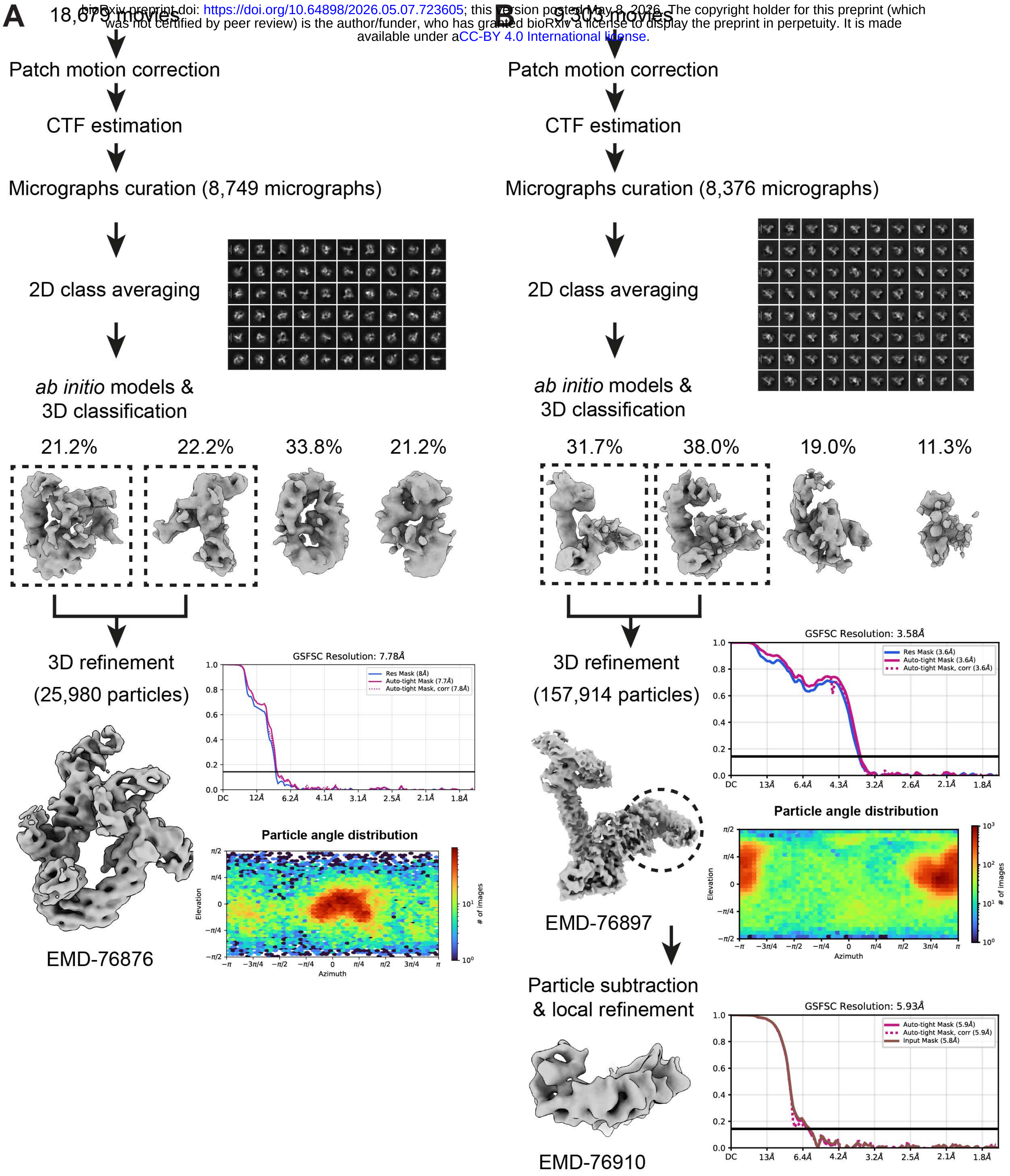

Figure S4

bioRxiv preprint doi: https://doi.org/10.64898/2026.05.07.723605; this version posted May 8, 2026. The copyright holder for this preprint (which was not certified by peer review) is the author/funder, who has granted bioRxiv a license to display the preprint in perpetuity. It is made available under aCC-BY 4.0 International license.

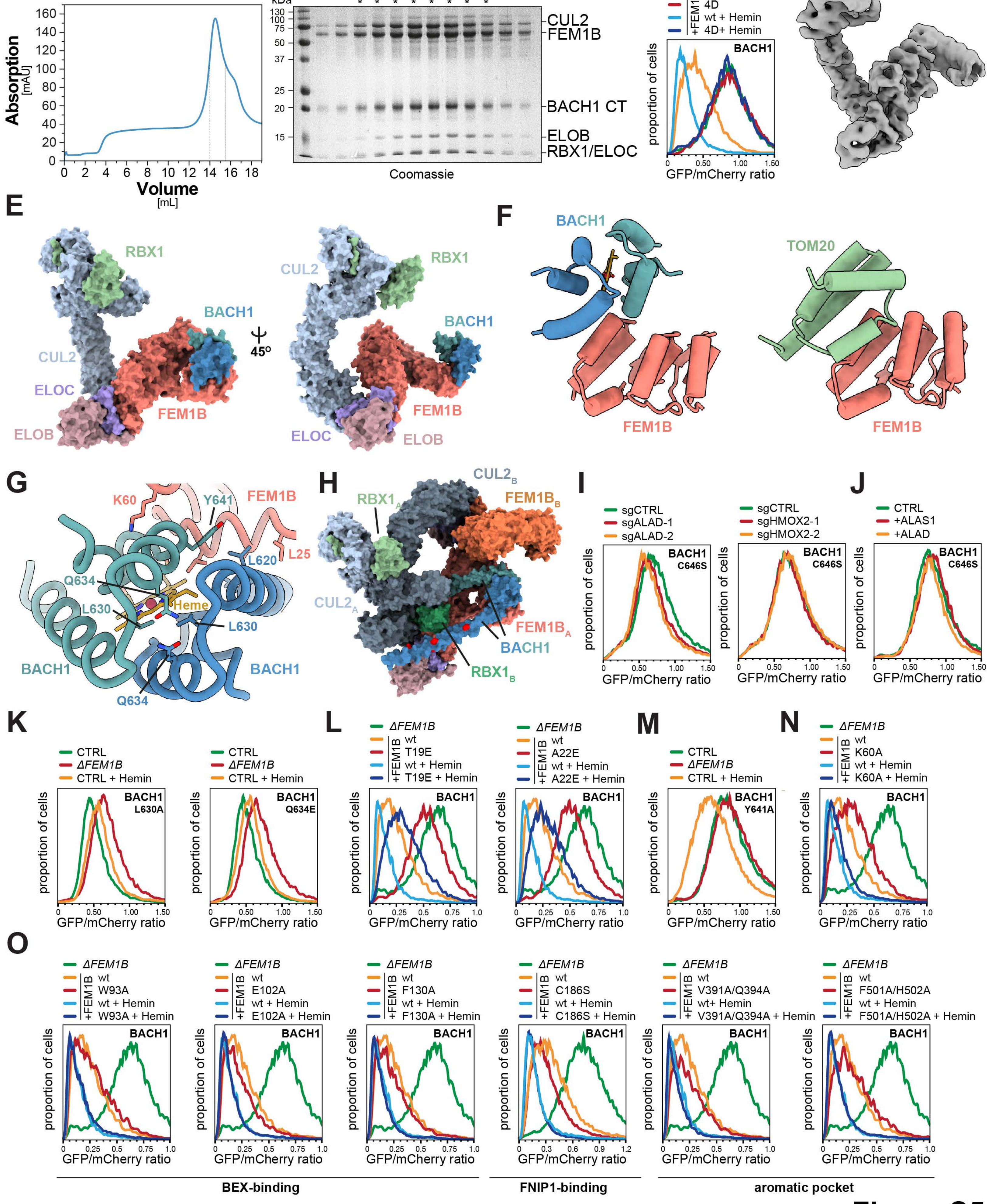

Figure S5

bioRxiv preprint doi: https://doi.org/10.64898/2026.05.07.723605; this version posted May 8, 2026. The copyright holder for this preprint (which was not certified by peer review) is the author/funder, who has granted bioRxiv a license to display the preprint in perpetuity. It is made available under aCC-BY 4.0 International license.

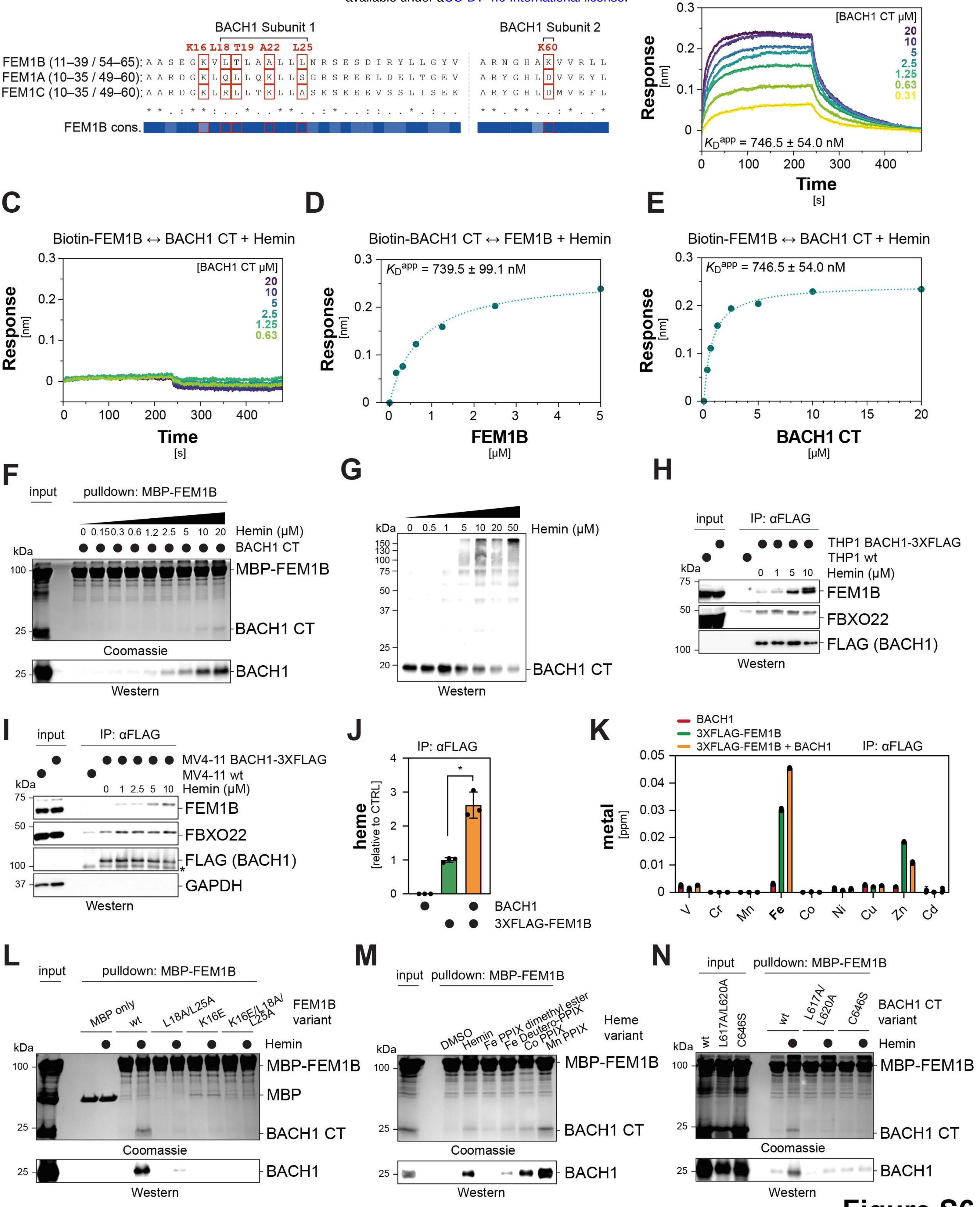

Figure S6

bioRxiv preprint doi: https://doi.org/10.64898/2026.05.07.723605; this version posted May 8, 2026. The copyright holder for this preprint (which was not certified by peer review) is the author/funder, who has granted bioRxiv a license to display the preprint in perpetuity. It is made available under aCC-BY 4.0 International license.

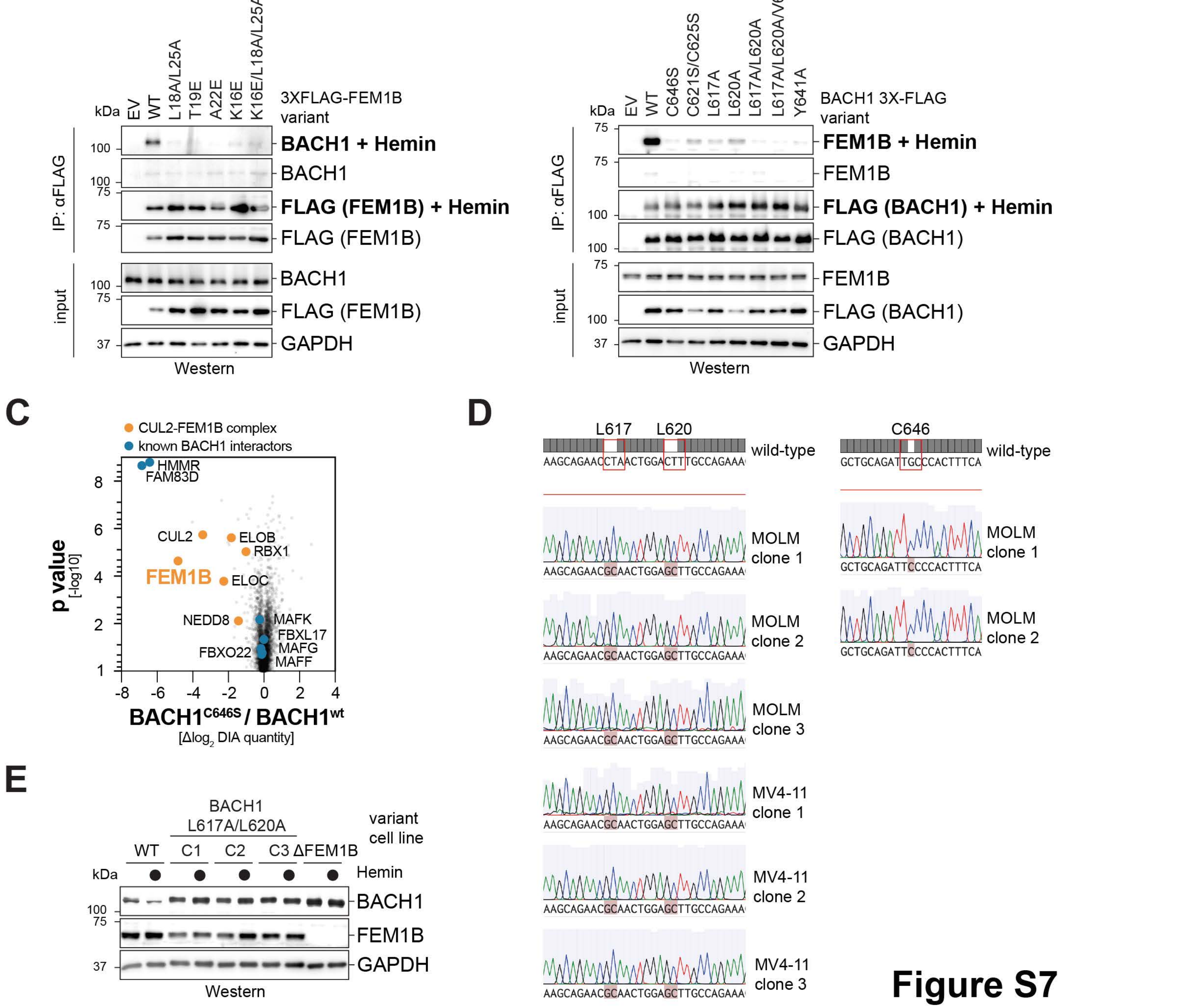

Figure S7

bioRxiv preprint doi: https://doi.org/10.64898/2026.05.07.723605; this version posted May 8, 2026. The copyright holder for this preprint (which was not certified by peer review) is the author/funder, who has granted bioRxiv a license to display the preprint in perpetuity. It is made available under aCC-BY 4.0 International license.

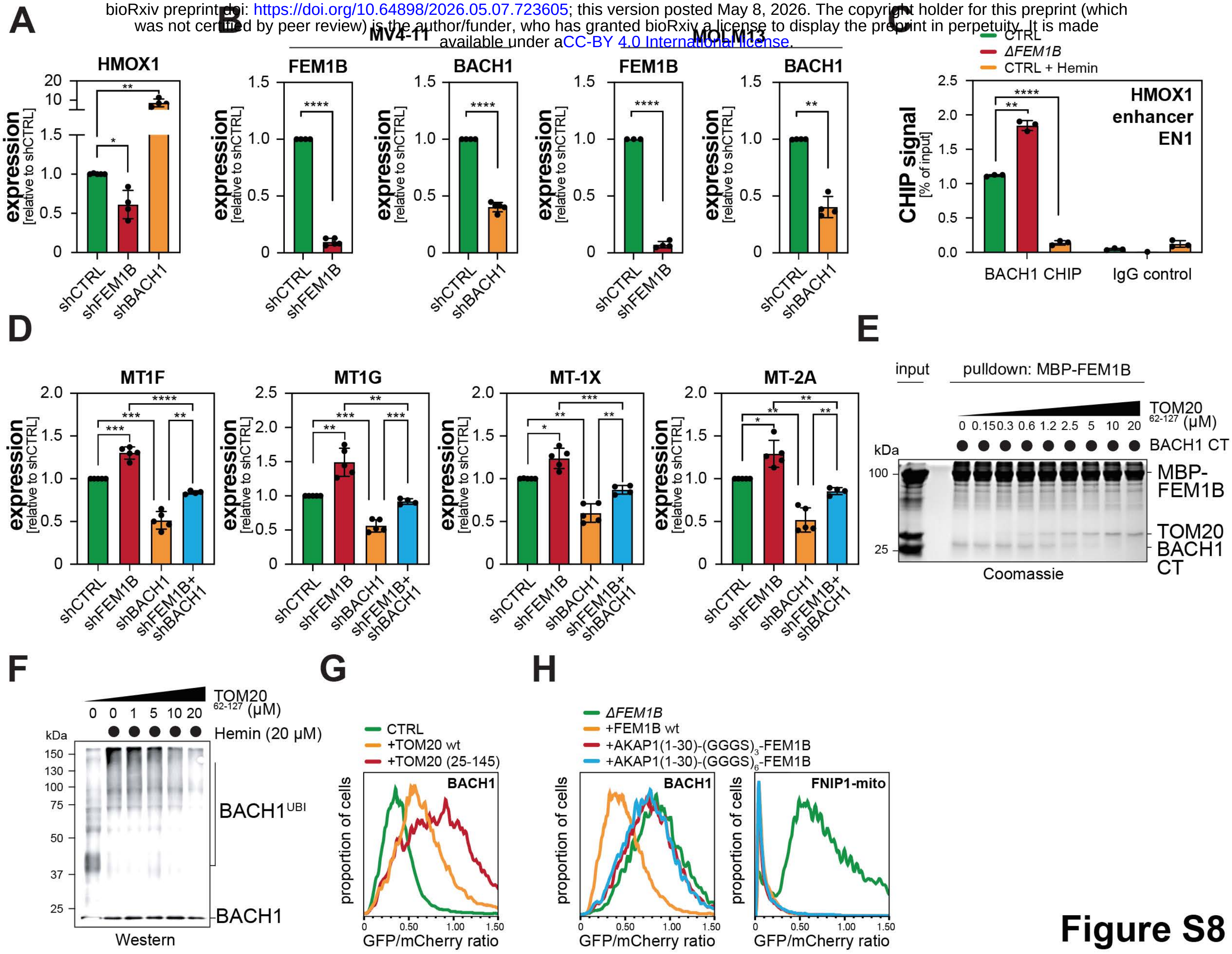

Figure S8

bioRxiv preprint doi: https://doi.org/10.64898/2026.05.07.723605; this version posted May 8, 2026. The copyright holder for this preprint (which was not certified by peer review) is the author/funder, who has granted bioRxiv a license to display the preprint in perpetuity. It is made available under aCC-BY 4.0 International license.

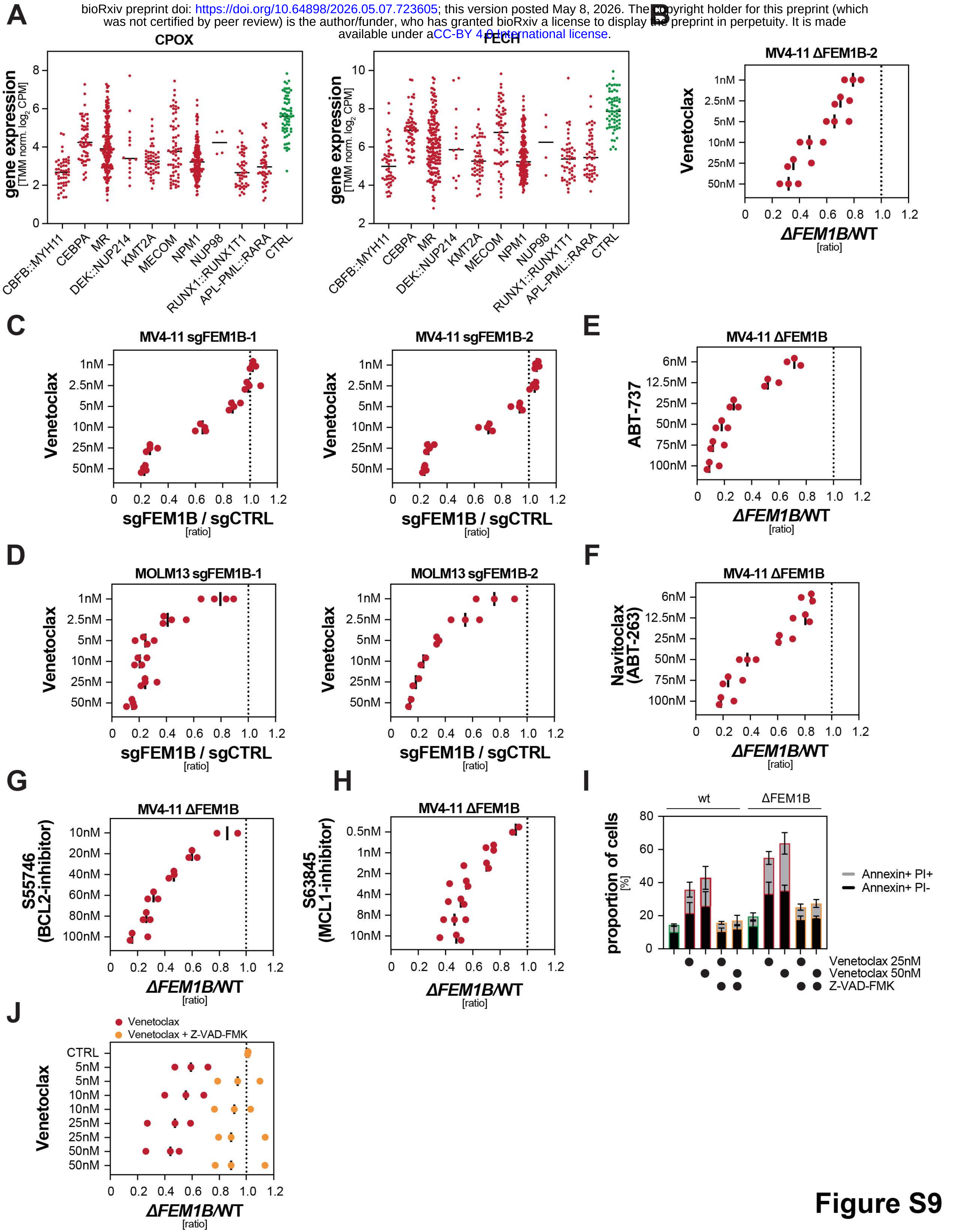

Figure S9
